# The diagnostic accuracy of spirometry as screening tool for adult patients with a benign subglottic stenosis

**DOI:** 10.1186/s12890-023-02592-4

**Published:** 2023-08-28

**Authors:** Juliëtta H.C. Schuering, Ilan J. Y. Halperin, Maarten K. Ninaber, Luuk N.A. Willems, Peter Paul G. van Benthem, Elisabeth V. Sjögren, Antonius P.M. Langeveld

**Affiliations:** 1 Department of Otorhinolaryngology and Head & Neck Surgery, Leiden University Medical Center Leiden, Zuid-Holland, The Netherlands; 2 Department of lung medicine, Leiden University Medical Center Leiden, Zuid-Holland, The Netherlands

**Keywords:** Spirometry, Expiratory Disproportion Index, Asthma, Subglottic stenosis

## Abstract

**Background:**

There is a considerable diagnostic delay in the diagnosis ‘benign acquired subglottic stenosis in adults’ (SGS, diagnosed by the reference standard, i.e. laryngo- or bronchoscopy). Patients are frequently misdiagnosed since symptoms of this rare disease may mimic symptoms of ‘asthma.’ The ‘Expiratory Disproportion Index’ (EDI) obtained by spirometry, may be a simple instrument to detect an SGS-patient. The aim of this study was to evaluate the diagnostic accuracy of the EDI in differentiating SGS patients from asthma patients.

**Methods:**

We calculated the EDI from spirometry results of all SGS-patients in the Leiden University Medical Center (LUMC), who had not received treatment 2 years before their spirometry examination. We compared these EDI results with the EDI results of all true asthma patients between 2011 and 2019, who underwent a bronchoscopy (exclusion of SGS by laryngo- or bronchoscopy).

**Results:**

Fifty patients with SGS and 32 true asthma patients were included. Median and IQR ranges of the EDI for SGS and asthma patients were 67.10 (54.33–79.18) and 37.94 (32.41–44.63), respectively. Area under the curve (ROC) of the accuracy of the EDI at discriminating SGS and asthma patients was 0.92 (95% CI = 0.86–0.98). The best cut-off point for the EDI was > 48 (i.e. possible upper airway obstruction), with a sensitivity of 88.0%% (95%CI = 77.2-95.0%%) and specificity of 84.4% (95%CI = 69.4-94.1%).

**Conclusions:**

The EDI has a good diagnostic accuracy discriminating subglottic stenosis patients from asthma patients, when compared to the reference standard. This measurement from spirometry may potentially shorten the diagnostic delay of SGS patients. Further studies are needed to evaluate clinical reproducibility.

## Background

An acquired benign subglottic stenosis in adults (SGS) is a rare condition that causes airway obstruction and may lead to severe respiratory distress. In a SGS, the region between the inferior arcuate line of the vocal cords and the lower margin of the cricoid is narrowed by excessive fibrosis [1, 2]. Etiological triggers for the development of this excessive fibrosis are auto-immune disorders (e.g. granulomatosis polyangiitis), traumatic or post-intubation/post-tracheostomy injuries to the airway, or sometimes unknown (idiopathic subglottic stenosis) [2, 3].

SGS-patients present with symptoms of dyspnea on exertion, shortness of breath, stridor, cough and sometimes change of voice [4]. These symptoms may resemble the symptoms of the more common diagnosis of asthma. Moreover, 33–37% of all subglottic stenosis patients are initially misdiagnosed with asthma [5–7]. This results in a mean diagnostic delay of 2 years, especially in idiopathic subglottic patients [5].

The gold standard instrument to diagnose an SGS, is endoscopy of the upper airway (laryngo- or bronchoscopy). Since a laryngo- or bronchoscopy is not part of the routine work-up of asthma patients, SGS can be missed easily. However, lung function testing including spirometry is standard of care for a patient presenting with dyspnea symptoms. Therefore, the diagnostic delay could potentially be shortened if the diagnosis of SGS could be made with spirometry. The flow-volume (FV) curves derived from standard spirometry, have long been described as a tool for detecting intra- or extra thoracal fixed upper airway obstructions [8, 9]. In patients with upper airway obstructions such as SGS, particularly inspiration is affected [8, 9]. This is reflected by the shape of the inspiration curve [8, 9]. However, even with optimal coaching it’s hard for patients to perform a reproducible forced inspiratory volume curve [10–12]. Measurement of the forced expiratory flow volume curve is the mainstay for diagnosing chronic obstructive lung disease and quality standards for its measurement have been well established and updated [12]. Therefore, an expiratory curve-derived screening parameter was proposed to detect an upper airway stenosis by Empey et al. [10]. Nouraei et al. validated this screening parameter further in 2013. They presented the ‘Expiratory Disproportion Index’ (EDI), containing the ratio of the expiratory curve derived parameters FEV_1_ (Forced Expiratory Volume in 1 s) and PEFR (Peak Expiratory Flow rate) [13]. This index is based on the alteration in the flow-volume curve if an upper airways obstruction is present [10]. When upper airways obstruction is present, the reduction in PEFR will be greater than the reduction in FEV_1_ [10]. The FEV_1_ measurement is obtained from the changes in maximal flow over a whole range of lung volumes and is hence much less dependent on effort and upper airways resistance than the PEFR [10].

Nouraei et al. found a sensitivity and specificity of the EDI in differentiating between laryngotracheal stenosis patients and non-stenosis patients of 95.9% and 94.2%, respectively [13]. Their non-stenosis group consisted of both lung patients and healthy subjects. They concluded that the EDI was exclusively elevated in upper airway obstruction patients and is therefore an excellent screening parameter for these patients [13]. However, in none of these non-stenosis patients endoscopy was performed as reference test. The presence of a stenosis was therefore not excluded in the non- stenosis cases.

Therefore, the aim of this study was to assess the diagnostic test accuracy of the EDI in differentiating between SGS patients and true asthma patients.

## Methods

### Design

In this retrospective diagnostic accuracy study, the EDI-values of all consecutive SGS patients (target condition) who visited our ENT department from 2011, were compared with the EDI-values of all consecutive asthma patients who presented to the lung department from 2011. The EDI values (index test) were calculated from a spirometry test within approximately 6 months prior or after laryngo- or bronchoscopy (reference test). Every patient had undergone a laryngo- or bronchoscopy of the upper airway as reference test, to confirm or rule out the presence of an upper airway obstruction. This study has been granted an exemption from requiring ethics approval by the local ethical committee; “Medisch-Ethische Toetsingscommissie Leiden-Den Haag-Delft.” All methods were performed in accordance with the relevant guidelines and regulations.

### Subjects

All consecutive patients with an SGS treated in our center since 2011 were identified. The SGS was confirmed by laryngo- or bronchoscopy. The exclusion criteria were SGS due to a head or neck malignancy or of caustic origin with lower airway involvement (e.g. ingestion of acidic liquids) or co-existing asthma or COPD to avoid bias by comorbidity. SGS patients were also excluded if they had undergone surgical treatment two years prior to a flow-volume loop measurement, to avoid measuring treatment follow-up effects such as postoperative edema or granulomas.

The asthma group included all adult patients (aged ≥ 18) who visited the outpatient lung clinic with the diagnosis asthma in our center between 2011 and 2019 and underwent both spirometry and bronchoscopy. This database was obtained, collecting the combination of spirometry and bronchoscopy activities in patients of the lung department in the Dutch version of the Diagnosis Related Group registration (DRG) of our center. Asthma diagnoses were confirmed by their treating pulmonologist. We excluded all asthma patients who had abnormalities in the upper airway discovered during their bronchoscopy and patients with malignancies in their medical history. Therefore, we only included “true” asthma cases.

### Data collection

Demographic data about age, sex, and diagnosis were collected. Lung function testing was done according to ERS standard of spirometry, using data from the flow-volume loop. They were derived with Jaeger^TM^CareFusion spirometry software.The forced expiratory volume in 1 s (FEV_1_) and peak expiratory flow rate (PEFR) were obtained. The EDI was calculated by the ratio of FEV_1_ (expressed in liters) to PEFR (expressed in liters per second) multiplied by 100 ($EDI=\left(\frac{FEV1}{PEFR}\right)*100$) [13]. In case of multiple spirometry tests taken around the bronchoscopy or laryngoscopy, the first sufficiently interpretable result was used.

### Analysis

The appropriate statistical tests concerning outcome variables were chosen after the distribution of all variables was analyzed. Receiver operating characteristic (ROC) statistics were used and graphed to determine the differentiating value of the EDI between SGS-patients and non-SGS patients. This allowed a comparison of the test accuracy of the new test (EDI) compared to the golden standard (a laryngo- or bronchoscopy) to detect an SGS. The optimal EDI cut-off point was chosen by analyzing the sensitivity and specificity with 95% confidence intervals for each EDI cut-off point using IBM SPSS statistics (version 25). The violinplot was conducted in R (R studio, 2009) using the package ggplot2.

## Results

### Demographics

82 patients (50 SGS and 32 asthma patients) were included in this study. Group characteristics are shown in Table 1. No significant differences were found between the SGS and asthma patients. There was a relatively high percentage of females in the SGS group (n = 39, 78.0%). However, this could be explained by the predominant amount of idiopathic stenosis in our SGS group (n = 27, 54.0%), which is predominantly seen in females.

There were various indications for a bronchoscopy in the asthma patients, but no structural airway abnormalities (e.g. subglottic stenosis) were encountered in any asthma patient. The indications for bronchoscopy in the asthma patients were: treatment resistant chronic coughing (n = 11), infiltrative or soft tissue abnormalities on radiographic examination (e.g. susceptive for tumors) (n = 6), bronchoalveolar lavage, biopsy or culture (n = 4), chronic cough with hemoptysis (n = 2), asthma exacerbation (n = 2), suspected lower airway abnormalities (n = 1) and atelectasis of unknown origin (n = 1). In 3 patients, there was suspicion of an upper airway abnormality due to the presence of an inspiratory stridor. However, bronchoscopy revealed no such cause in all 3 patients.

**Table 1:** Demographics

	SGS patients (n = 50)	Asthma patients (n = 32) *p*-values
Female (n, %)	39 (78.0%)	21 (65.6%) *p =* 0.349
Age at spirometry test date in years (median, IQR)	48.0 (38.5–60.3)	51.0 (39.5–66.5) *p =* 0.655
BMI at spirometry test date (median, IQR)	26.1 (23.9–28.8)	26.7 (22.7–30.9) *p* = 0.766
Stenosis etiology (n, %)		
Idiopathic	27 (54.0%)	
GPA	13 (26.0%)	
Amyloidosis	1 (2.0%)	
Sarcoidosis	1 (2.0%)	
Post-intubation	3 (6.0%)	
Post-tracheotomy	4 (8.0%)	
Post traumatic	1 (2.0%)	
Asthma etiology (n, %)		
Allergic		11 (34.4%)
Non-allergic		17 (53.1%)
Allergic bronchopulmonary aspergillosis		4 (12.5%)
Spirometry variables (median, IQR) - %pred. value (median %)		
FEV1 in liters	2.57 (2.06–3.14) – 86.0%	2.22 (1.78–3.09) – 83.0% *p* = 0.61
PEFR in liters/second	3.81 (3.15–5.20) – 60.6%	6.04(4.43–8.22) – 91.5% *p* = 0.00^*^
FVC in liters	3.83 (3.23–4.37) – 101.5%	3.31 (2.61–4.09) – 95.5% *p* = 0.06
VC in liters	3.33 (2.83–3.96) – 102.0%	3.88 (3.31–4.37) – 97.0% *p* = 0.14

Demographic data of the “benign subglottic stenosis”(SGS) patients and the ‘asthma’ patients. GPA = Granulomatosis with polyangiitis (Wegener’s granulomatosis); FEV1 = Functional expiratory volume after 1 second; PEFR = Peak expiratory flow rate; FVC = Forced Vital Capacity; VC = Vital capacity. % pred. value = % predicted value of spirometry variables. GLI-2012 was used to as reference value for the pred. values [11]. P-values correspond to the differences testing between groups

### EDI results

**Fig. 1:**
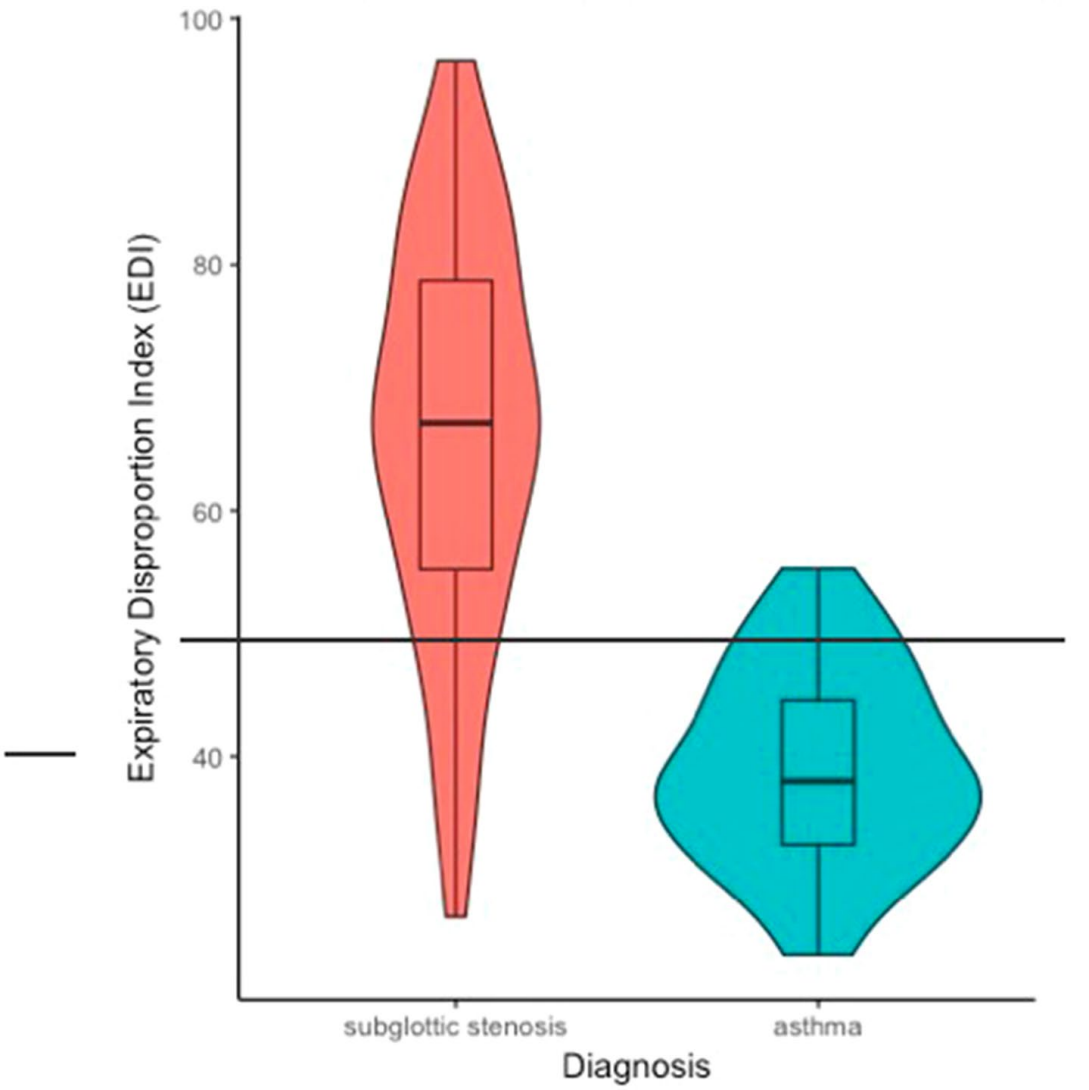
The range of EDI values in the subglottic stenosis group(red) was 26.93–96.57, while in the asthma group (blue) EDI values ranged 23.83–55.26. The ideal EDI cut-off point was set at 48

**Fig. 2:**
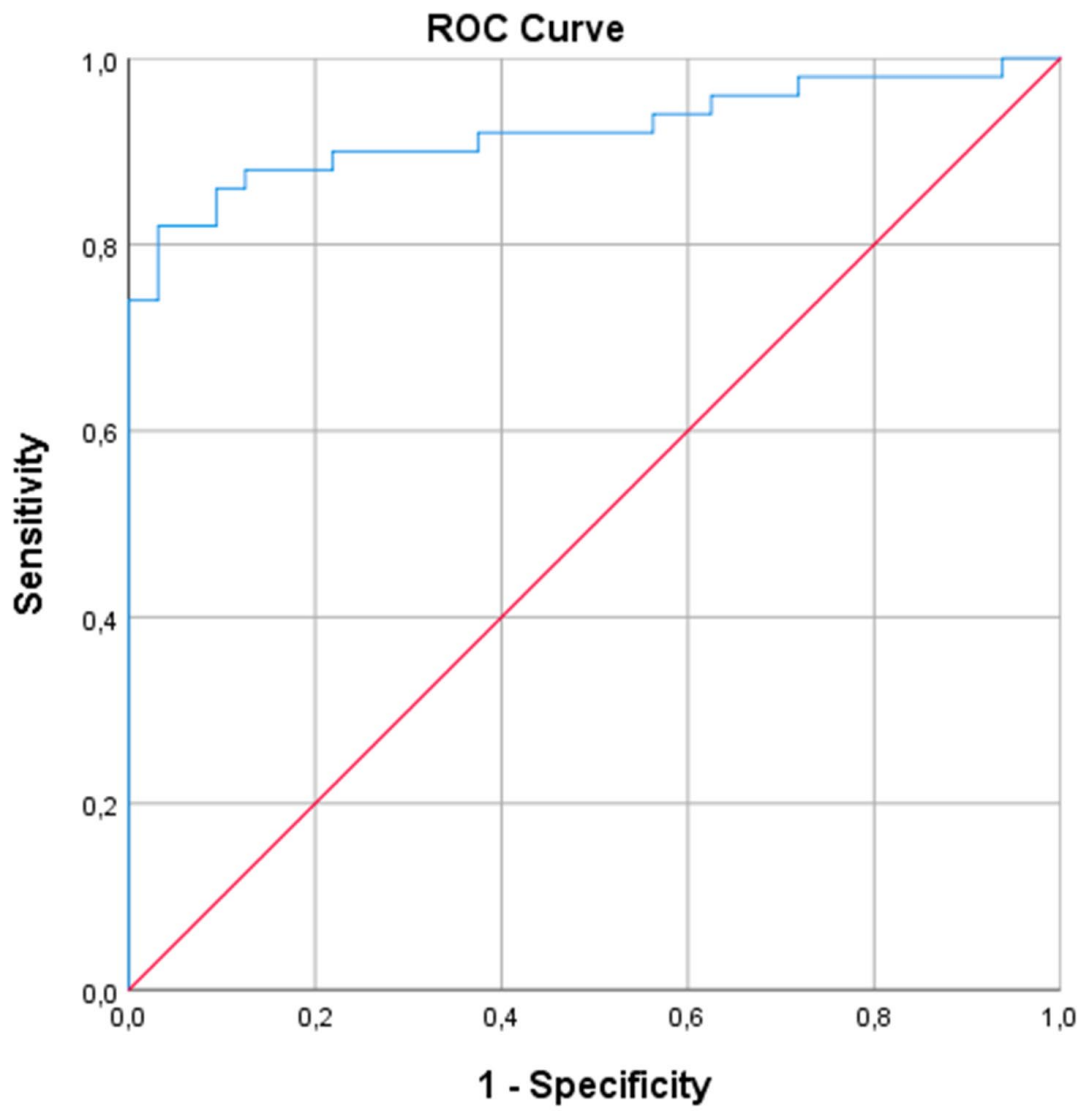
**ROC-curve Expiratory Disproportion Index (EDI)** ROC-curve for EDI results. Area under the curve (AUC) = 0.92 (95% CI = 0.86–0.98), implying a good to excellent accuracy of the EDI at discriminating SGS and asthma patients

A violin plot of the EDI values of both groups is shown in Fig. 1. Median and IQR ranges for SGS and asthma patients were 67.10 (54.33–79.18) and 37.94 (32.41–44.63), respectively. The area under the curve (AUC) in the ROC curve (Fig. 2) was 0.92 (95% CI = 0.86–0.98), implying a good to excellent accuracy of the EDI at discriminating SGS and asthma patients. An EDI > 50 as cut-off point showed a sensitivity of 86.0% and specificity of 90.6%. However, the best cut-off point for the EDI was found at an EDI > 48 (i.e. possible upper airway obstruction), which showed a sensitivity of 88.0%% (95%CI = 77.2-95.0%%) and specificity of 84.4% (95%CI = 69.4-94.1%).

Table 2 presents the 2 × 2 contingency table for these results. There were 6 SGS patients with an EDI of < 48. Their EDI values were 26.93, 35.14, 36.06, 37.20, 40.64 and 45.68. Five out of the 6 patients had their SGS due to GPA and had concomitant pulmonary GPA-activity, which caused obstructive lung disease. The 6th patient had a post-tracheostomy stenosis without additional lung disease. These 6 SGS patients with an EDI of < 48 would initially have been missed if indication for laryngoscopy was solely based on spirometry results. In the asthma group, 5 patients had an EDI of > 48. There were no specific remarkable disease or spirometry details in these patients.

**Table 2:** 2 × 2 contingency table: Expiratory Disproportion Index (EDI)

	SGS patients	Asthma patient	Total
EDI > 48	44	5	49
EDI < 48	6	27	33
Total	50	32	82

Table 2. Expiratory Disproportion Index (EDI) results for all 82 patients with subglottic stenosis (SGS) of asthma and a cut-off point of EDI > 48. An EDI > 48 would mean a referral for a laryngoscopy, < 48 would mean no referral for a laryngoscopy

## Discussion

The current study indicates that the Expiratory Disproportion Index (EDI) is a sensitive and specific parameter to discriminate between subglottic stenosis and true asthma patients in a well-defined study population. These results strengthen the validation and the expected value of the EDI as screening tool for subglottic stenosis.

Today, there’s a lack of evidence on the validity of the EDI as screening parameter for upper airway stenosis. To our knowledge, there are only two recent studies who reported on the diagnostic accuracy of the EDI for upper airway stenosis patients: Nouraei et al. in 2013 and Calamari et al. in 2020 [13, 14].

Nouraei et al. reported a sensitivity of 95.9% (95%CI = 92.7–97.0%) and specificity of 94.2% (95%CI = 93.6–94.0%) of the EDI, to discriminate between upper airway stenosis and non-stenosic patients. The results in our study showed a sensitivity of 88.0% (95%CI = 77.2–95.0%) and a specificity of 84.4% (95%CI = 69.4–94.1%) for the EDI to discriminate between stenosis and asthma patients. The bootstrap analysis in our study resulted in a slightly different cut-off point of an EDI > 48 indicative for upper airway stenosis, compared to Nouraei et al. who reported EDI > 50 as cut-off point.

However, just as in the study of Nouraei et al., our study confirmed the great differentiating abilities of the EDI in screening between SGS and asthma patients. The small difference in sensitivity, specificity and cut-off point could be explained by differences in study populations. In contrast to Nouraei et al., we included only one EDI result of each unique SGS patient. The accuracy of the EDI might be slightly higher using repeated measurements within the same patient, as Nouraei et al. did (they included 217 EDI results from 156 unique laryngotracheal stenosis patients) [15]. Furthermore, in contrast to Nouraei et al., we focused on evaluating the diagnostic test accuracy value of the EDI solely for benign etiologies of SGS patients, because the diagnostic delay is a clinically relevant problem, especially in this group. Finally, unlike Nouraei et al., our study validated the EDI as index test using only “true” SGS and “true” asthma patients. This could also have affected the diagnostic accuracy parameters of Nouraei et al., since they did not rule out the presence of an upper airway obstruction in their non-stenosis group by laryngo- or bronchoscopy .

The second report on the EDI, of Calamari et al. in 2020, focused on the influence of obesity on the diagnostic accuracy of the EDI as screening tool for upper airway stenosis [14]. They defined obesity as a BMI > 30 [14]. They found a sensitivity of 50.0% (95% CI 32.7%-67.3%) and specificity of 71.9% (95% CI 68.2%‐75.6%) using a cut-off EDI value of > 50 for obese stenotic versus obese non-stenotic patients [14]. They concluded that although the mean EDI values were significantly different in stenotic and nonstenotic patients in both BMI cohorts, the EDI was not as sensitive at identifying stenotic cases in obese patients as in nonobese patients [14]. Our study cohort didn’t include just a few obese patientsso it was not possible to confirm this. However, the findings of Calamari et al., do suggest that the presence of comorbidities affecting lung function (e.g. obesity) influence the diagnostic accuracy of the EDI for a upper airway stenosis. An illustrative finding of this concept in our study, is that 5 of our 6 SGS patients with an EDI < 48, demonstrated affected lung function by concomitant obstructive GPA lung disease. Previous literature showed that the EDI is also low in people with lung diseases such as COPD or pulmonary fibrosis, respectively EDI 34.1 versus 34. However, it is uncertain what the effect of comorbidity is on the EDI. Althoughstudy numbers are small and caution should be taken when drawing conclusions, it might be possible that the EDI is not able detect a SGS in patients with extensive coexisting pulmonary disease.

To our knowledge, this is the first study to test the diagnostic accuracy of the EDI by comparing it to the standard reference test (laryngo- or bronchoscopy) in all patients. The fact that we evaluated only ‘true SGS’ and ‘true asthma patients’ strengthens the internal validity of this study.

Finally, we only used the spirometry results before treatment (or > 2 years after last treatment for SGS patients) in all SGS cases. This resembled the EDI values ​​from the SGS patient at first presentation and thereby approximated the target patient population where the EDI should eventually be implemented to reduce the diagnostic delay.

This diagnostic accuracy study also had some limitations. Firstly, it only included asthma patients who underwent a bronchoscopy, which is unusual care for most asthma patients. It is unclear whether this affected our results, although we suspect there is no reason to believe that our spirometry results differ much from those of other asthmatics. Our EDI values ​​were consistent with those of asthmatics from previous studies. This also applied to our SGS patients. Also the SGS EDI values in our study were roughly in line with previously reported EDI values of upper airway stenosis patients. Our SGS patients had a median EDI of 67.10 (54.33–79.18). This was comparable to SGS-EDI values described in literature. Nouraei et al. found a mean EDI of 76 ± 17 in benign laryngotracheal stenosis cases [13]. Tie et al. described a mean preoperative EDI of 81.3 ± 16.0 in 12 idiopathic SGS patients [16]. Carpenter et al. evaluated the preoperative EDI values of 42 idiopathic SGS patients, stratified by stenosis grade, with use of the Cotton-Meyer grading system [17].They found mean (95%CI) EDI values for stenosis grade I, II and III of 44.6 (41.7–47.5), 62.6 (58.2–66.1) and 77.9 (73.2–82.5), respectively [17]. Our asthma patients had a median EDI of 37.94 (32.4–44.6). This was comparable with the mean EDI of 36.9 (± 7.9) in 1600 asthma patients, described by Noureai et al.[15].

Secondly, data on fiberscope findings and stenosis severity, which is usually graded with the Cotton-Meyer scoring system, were not collected [18]. Therefore, we could not stratify our results based on stenosis severity. However, we argue that this grading system is of limited value in the assessment of stenosis severity, since it is very suspectable to interobserver variability, as a slight alteration in the viewing angle of the fiberscope alters the accuracy of this estimation [19–21]. For defining subtle differences in severity of a stenosis (which is sometimes a difference of only millimeters) or during treatment follow-up, the EDI is not specific enough. The EDI is based on expiratory parameters and especially the inspiratory parameters are affected in stenosis patients. Therefore, during follow-up we suggest using the Are Under the Curve (AUC) of both the expiratory- as the inspiratory loop) [22]. Finally, our study focused on a select small group of SGS and asthma patients, in an academic tertiary care center. We excluded SGS patients with coexisting lower airway disease (other than GPA). This means that these results do not directly reflect the heterogeneity of daily clinical practice and disregard the potential influence of coexisting obstructive pulmonary diseases on the EDI accuracy.

This study emphasizes that the EDI is a reliable screening parameter for SGS, something that is much needed in this diagnosis. The most important advantage of the EDI, is that it is a non-invasive and immediately implementable derivative of standard spirometry. Especially, considering spirometry is standard of care in the target population where the EDI must be implemented to shorten the doctor’s delay (the asthma patient clinic).

The next step in the evaluation of this potential new diagnostic screening tool, is to assess its external validity by generalizing the findings in a heterogeneous group [21]. Currently, we are conducting the next step of this validation process by evaluating the accuracy of the EDI in a more heterogenous study population, representing the daily clinical practice.

## Conclusion

In this diagnostic accuracy study, we have shown that the EDI has an excellent sensitivity and specificity in discriminating subglottic stenosis patients from asthma patients when compared to laryngo-bronchoscopy as gold reference standard. This confirms and strengthens previous findings. Further studies are needed to evaluate the reproducibility in an open care setting.

## Data Availability

All datasets used and/or analysed during the current study aravailable from the corresponding author on reasonable request.
